# Heterogeneity in grocery shopping patterns among low-income minority women in public housing

**DOI:** 10.1186/s12889-022-14003-0

**Published:** 2022-08-24

**Authors:** Victoria Shier, Sydney Miller, Ashlesha Datar

**Affiliations:** 1 Price School of Public Policy, Schaeffer Center for Health Policy and Economics, University of Southern California, 635 Downey Way, Los Angeles, CA 90089 USA; 2 Department of Population and Public Health Sciences, University of Southern California, 2001 N Soto St, Los Angeles, CA 90033 USA; 3 Center for Economic and Social Research, University of Southern California, 635 Downey Way, Los Angeles, CA 90089 USA

**Keywords:** Food access, Grocery shopping, Public housing, Low-income women

## Abstract

**Background:**

Public housing residents, who tend to be predominantly female and racial/ethnic minorities, are at a particularly high risk for chronic health conditions. Prior studies have suggested that a lack of access to healthy and affordable food may be an important barrier in public housing communities, but evidence is mixed on the association between the neighborhood food environment and dietary quality, suggesting the need to examine food access patterns in low-income, minority communities more deeply. The purpose of this study was to examine the variability in grocery shopping patterns, and the factors that predict them, among low-income minority women in public housing.

**Methods:**

Interviewer-administered surveys and body composition measurements were collected in the Watts Neighborhood Health Study, an ongoing longitudinal cohort study of low-income urban public housing residents located in South Los Angeles. Descriptive analyses were conducted to understand the variation in grocery shopping patterns among women. Logistic and ordered logistic regression models were estimated to examine the association between resident characteristics and grocery shopping patterns.

**Results:**

There was considerable variability in grocery shopping patterns, including the types of grocery stores accessed, distance travelled, frequency of shopping, and reasons behind grocery store choice. Grocery shopping patterns were associated with several participant characteristics, including race/ethnicity, working status, access to a car, income, and education. Hispanic participants were less likely to shop at a supermarket, travel further distances to shop, shop more frequently, and were more likely to prioritize price in their choice of primary grocery store than non-Hispanic Black women participants.

**Conclusions:**

There was considerable variability in grocery shopping patterns, even *within* this low-income, minority community despite access to the same neighborhood food environment. Convenience and quality, in addition to price, were priorities for choice of primary grocery store, and differences by race/ethnicity suggest that initiatives to improve the neighborhood food environment should consider quality of food, cultural factors, and availability of foods desired by the surrounding community, in addition to price and proximity of grocery stores.

**Supplementary Information:**

The online version contains supplementary material available at 10.1186/s12889-022-14003-0.

## Background

Low-income and minority populations are at an increased risk for unhealthy dietary patterns [1–4] and diet-related diseases, including obesity and cardiovascular disease [5–8]. Disparities in diet and disease are driven by complex, multilevel factors, including the economic, social, and built environments in which individuals access and consume food [9]. One factor that has been linked with these disparities is the neighborhood food environment. Minority and low-income individuals are more likely to live in areas with limited supermarkets and grocery stores [10–12] and predominantly small food outlets that have more expensive and less quality healthy foods [12–14].

Although some studies have indicated that decreased access to healthy grocery stores in one’s neighborhood is associated with poor dietary quality, others have not found any link [11, 12, 15–19]. This mixed evidence may be due to significant variation in grocery shopping patterns at the individual level, including choice of grocery shopping location. For example, previous studies have indicated that many individuals bypass their nearest food outlet [20–23] and shop for food at stores that are beyond the 1-mile radius, which is often used to define their neighborhood food environment [24]. Overall, shoppers at lower priced supermarkets are more likely to have lower education, lower income, and be older [21]. Within low-income communities, however, little is known about *why* individuals bypass their nearest food outlets, and why there is such variation in grocery shopping patterns. In particular, there is limited evidence on what individual and household characteristics may be associated with these patterns and other factors that may supersede proximity when deciding where to shop for groceries. Evidence from qualitative studies indicates that Hispanics travelled further to seek out tiendas or ethnic-specialty foods [25]. Additionally, low-income individuals have reported choosing grocery stores based off price [26]. For individuals living in low-income communities that are dominated by small, more expensive markets, it may be necessary to travel longer distances to find stores that fit within their budgets [26–30]. Consequently, they may also have to shop less frequently and trade out fresh foods for items that will last longer, such as boxed and canned foods [26, 29, 30], further exacerbating the risk for unhealthy diet. However, even when new, affordable grocery stores and supermarkets are introduced into low-income neighborhoods, many individuals still choose to shop at their usual outlet rather than the new store [31–33], and dietary patterns remain relatively unaltered [34, 35]. All this suggests that grocery shopping patterns may be more variable and complex.

This study adopts a Social-Ecological Framework, which emphasizes that individuals’ dietary behaviors are the result of complex, multilevel influences within one’s social and built environment, that interact reciprocally with individual behavior, demographics, and preferences [9]. The present study examines variability in grocery shopping patterns *within* low-income public housing communities in South Los Angeles. Public housing residents, who are predominantly female and racial/ethnic minorities, are at a particularly high risk for chronic health conditions [36–40]. Studies have suggested that a lack of access to healthy and affordable food may be an important barrier in public housing communities [27, 28, 41, 42], especially among those who are even further constrained by lack of personal transportation [23, 27]. An important feature of public housing communities is their clustered housing, which means all residents are exposed to the same food environments in their neighborhood. This creates a valuable opportunity to study variability in grocery shopping patterns, conditional on food environment. Specifically, the present study examines grocery shopping patterns including distance travelled to one’s primary grocery store, type of stores visited, and reasons for selecting a primary grocery store among minority women in public housing, and the individual and household characteristics that explain variability in these grocery shopping patterns. This is an important contribution because prior research typically treats low-income communities as a homogeneous group and comparisons are primarily made between socioeconomic groups [21, 43]. By focusing on variability in grocery shopping patterns *within* low-income households, our study can inform policies that seek to address the diverse needs of low-income families, instead of one-size-fits all approaches.

This study is also important because the community where our study sample is located recently initiated a large-scale redevelopment that will entail changes to the built environment, including addition of a new supermarket. Our study will allow us to benchmark grocery shopping patterns in this community prior to the redevelopment, which is the first step for understanding how grocery shopping patterns will change subsequently as a result of the redevelopment. Future analyses will examine the effect of the opening of the new supermarket on residents’ shopping patterns and diet.

## Methods

### Overview of Watts Neighborhood Health Study

The Watts Neighborhood Health Study (WNHS) is an ongoing longitudinal cohort study of low-income urban public housing residents located in the predominantly Latinx and Black community of Watts in South Los Angeles. The WNHS was designed to evaluate the impact of a large public housing redevelopment on residents’ obesity and related health behaviors. The redevelopment, which is occurring in Jordan Downs, involves major changes to the built and social environments in the community, which includes opening of a new supermarket, as well as brand new housing for the original residents. The study recruited adult and child residents from Jordan Downs, and two other public housing communities not undergoing redevelopment (Imperial Courts, Nickerson Gardens) in Watts during 2018–2019. The majority of participants were recruited from Jordan Downs due to study design consideration described elsewhere [44]. Participants were recruited through a multi-pronged approach collaborating with resident leaders at each site, including distribution of flyers and letters to homes, promotion at onsite events, and door-to-door visits. Data collection occurred at an on-site community space or the participant’s home. Participants completed an interviewer-administered survey about obesity-related behaviors and risk factors including dietary intake, physical activity, psychosocial risk factors related to diet and physical activity, health and well-being, home food environment, grocery shopping behaviors, and socio-demographics. Participants also completed body composition measurements conducted by trained study staff. Additional details on the methods of the overall study are presented in another paper [44].

### Study sample

Data used in the present study were collected during wave 1 (baseline) of the Watts Neighborhood Health Study. A total of 531 women consented and 507 had complete data for participant characteristics. There were 63 men who also participated but were excluded from this analysis due to small sample size to examine gender separately.

The study also conducted an audit of all businesses and storefronts, including the name, address, and type of business/organization, in Watts in 2018–2019. These data were used to describe the neighborhood food environment in Watts. The study was approved by the institutional review board at the University of Southern California.

### Measures

Adults were asked, “What is the name of the main store [and address] where your family does your major food shopping?” Using this information, we identified each household’s primary grocery store. We calculated the driving distance from each public housing site to each participant’s primary grocery store. We also classified each primary grocery store into three mutually exclusive categories: 1) Supermarket (chains with ≥ 10 locations; e.g., Food4Less); 2) Supermarket (non-chain with < 10 locations); 3) ethnic-specialty (e.g., Northgate Gonzalez Market); and 4) Warehouse clubs/Mass merchandiser-supercenter (e.g., Costco, Walmart Supercenter); 5) Other (convenience/mini markets,farmer’s market) [45].

Adults were also asked—(1) how many times their family visited the primary grocery store for major grocery shopping the past month, (2) how many other stores their family has visited for major grocery shopping in the past month (in addition to the primary grocery store), and (3) how many times their family has gotten food from a food pantry, food bank, local church or other agency that provides free groceries (community resources) in the past month.

Lastly, adults were asked: “If you were to choose just one main reason that your family does your major food shopping at this store, which would you choose? Is it the quality of the food, the price, the choice of items, the convenience of the location, the customer service, or the cleanliness?” We created indicator variables for the main reason the family shops at the primary grocery store based on the response to this question.

We examined the association between grocery shopping patterns and several explanatory variables that have been theoretically or empirically linked with grocery shopping behaviors in previous studies [9, 20–22]. We included whether the participant was 60 years old or older, whether household income was at or greater than $10,000, whether the participant’s highest level of education was High School (HS)/secondary school or higher, whether the participant works for pay, whether participant has access to a car, van, or truck when they need one, and whether participant has children younger than 18 years old living in the household. As lifestyle indicators and obesity status have been previously linked with shopping behaviors [21], we also included whether the participant was obese (based on anthropometric measurements including height and weight), and whether the participant rates their diet as very good or excellent. We also included an indicator for Jordan Downs, the site with the majority of participants and the primary focus of the redevelopment, to control for unobserved confounders that could be correlated with grocery shopping patterns.

### Statistical analysis

To examine the availability of food outlets and the location of where residents shopped, we used ArcGIS to create a map with: (1) the location of all food outlets (supermarkets, ethnic-specialty stores, and other such as small markets) within the Watts neighborhood (from the audit data), and (2) the location of all grocery stores where at least 5 residents in our sample shopped. Descriptive analyses were conducted to better understand the variation in grocery shopping patterns among study participants. Logistic and ordered logistic regression models (after testing the proportional odds assumption) were estimated to examine the association between resident characteristics and grocery shopping patterns, including whether the primary grocery store was a supermarket, distance to primary grocery store, number of times visited primary grocery store in past month, number of different grocery stores shopped at in past month, and number of times got food from community resources in the past month. Using logistic regression models, we estimated associations between the individual and household characteristics and the reasons for primary grocery store choice for the choices that were indicated by at least five percent of the sample. The potential presence of collinearity was assessed using variance inflation factor (VIF) < 4; no collinearity was detected. Sensitivity analyses were also conducted to assess the robustness of findings. We replicated the analyses using alternative explanatory variables (i.e. finer categories for age: 18–34, 35–59, and 60 + years old; household income: Less than $5,000, $5,000-$9,999, $10,000-$14,999, $15,000 or more; education: middle school/primary school orlless, some high school/secondary, high school/secondary/GED, more than high school/secondary; weight status: normal weight, overweight, and obese; and diet quality: poor or fair diet, good diet, very good or excellent diet). Robust standard errors were implemented in all analyses. Analyses were conducted using Stata 15.1 (StataCorp, College Station, TX).

## Results

### Descriptive analyses

Descriptive statistics of the sample (*N* = 507) are summarized in Table 1. Participants were all female and the majority were Hispanic (60.4%), had at least one child (65.5%), and were obese (64.1%). About half of the participants had a household income below $10,000 a year (50.7%). Most participants (65.5%) also reported that their diet was poor or fair.

**Table 1:** Description of sample

Sample *N* = 507	%
Age	
18–34	26.8
35–59	61.9
60 +	11.2
Race/ethnicity	
Hispanic	60.4
Non-Hispanic Black	38.3
Non-Hispanic Other	1.4
HH Income	
Less than $5,000	31.8
$5,000—$9,999	18.9
$10,000 – $14,999	17.6
$15,000 or more	31.8
Education	
Middle school /primary school or less	24.5
Some high school/secondary	22.7
High school/secondary/GED	31.8
More than high school/secondary	21.1
Working	32.2
Has access to car	77.1
Kids in household	
0 kids	33.5
1–2 kids	45.4
3 + kids	21.1
Obese	64.1
Healthiness of diet	
Poor	20.7
Fair	44.8
Good	20.7
Very good	7.7
Excellent	6.1

**Fig. 1:**
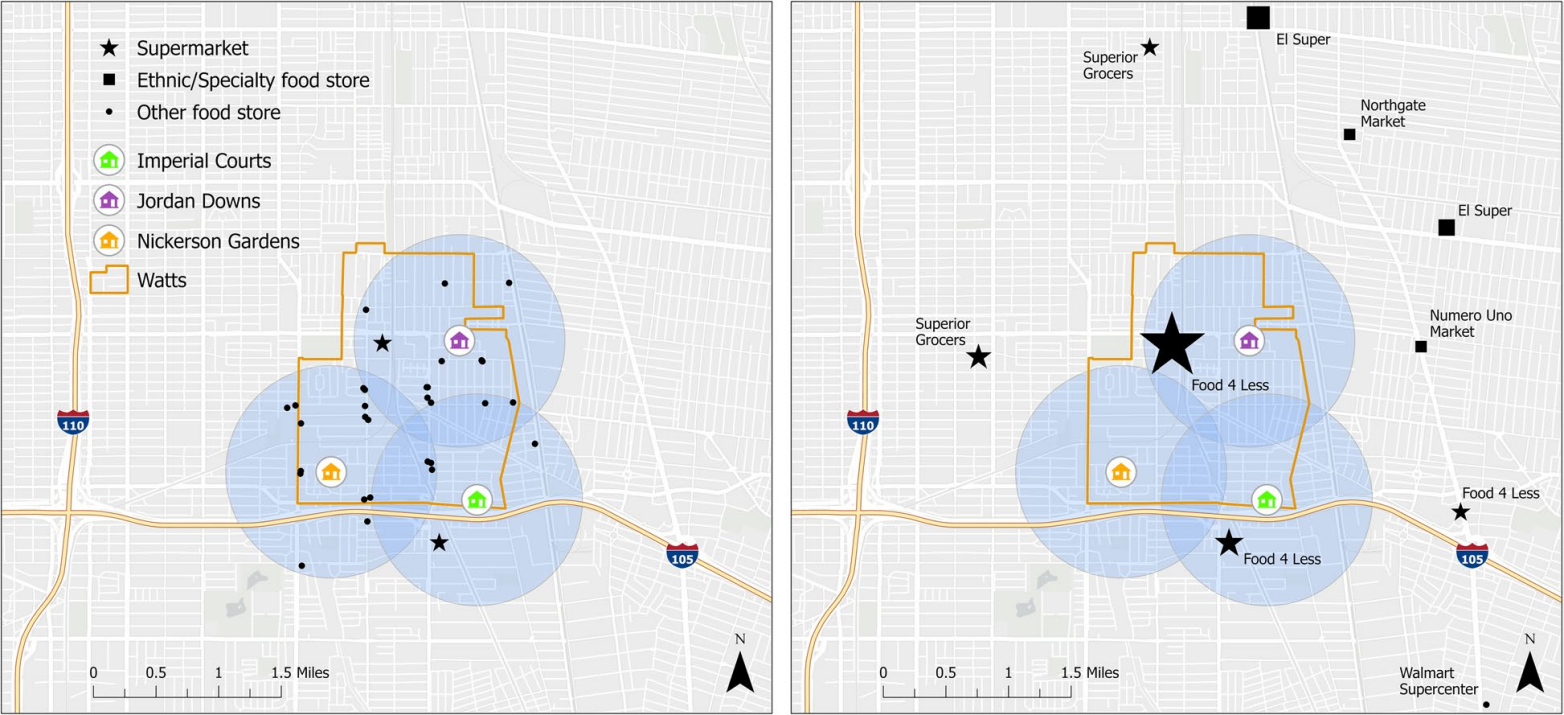
Food outlet availability (left) and primary grocery store (right) of public housing residents in Watts, LA

The availability and use of grocery shopping outlets is shown in Fig. 1. Figure 1 (left) maps all food outlets (including supermarkets, ethnic-specialty stores, and other such as small markets) surrounding the three public housing sites (with blue circles showing one half mile buffers from the public housing site borders). This figure shows that within one half mile of the public housing sites there are many ‘other’ food outlets, which were all small markets, convenience stores, or dollar stores. Two of the three sites have one supermarket and one site has no supermarket within one half mile. There were no ethnic-specialty stores within one half mile of any of the three public housing sites. Figure 1 (right) maps the primary grocery stores that at least five participants in the study shopped at, with larger icons representing more participants shopping at that location. There were 12 primary grocery stores where at least five participants in the sample shopped (two of these, Costco and Winco, are not shown in the figure because they are located more than eight miles from Watts). More than half of participants (54.7%) shopped at the two local supermarkets (Food4Less) and 28.9% shopped at the 10 other grocery stores (including supermarkets, ethnic-specialty stores, and other stores) where at least five participants shopped. The remaining participants (16.4%) shopped at more than 40 other grocery stores (not shown in figure).

Grocery shopping patterns are summarized in Table 2. Overall, most participants shopped at a supermarket (80.3%) or ethnic-specialty store (14.4%) for their primary grocery shopping (Table 2). There was considerable variability in grocery shopping patterns. Just about half of participants travelled less than one mile to their primary grocery store (50.6%), but 19.6% travelled more than three miles. One-third of participants reported that they visited their primary grocery store two to three times per month, but 12.0% reported zero to one time per month, and 26.0% reported six or more times. The majority (63.2%) of participants shopped at one or two stores, but a small percentage (13.4%) shopped at four or more stores. Just over a third of participants (35.9%) received food from community resources at least once in the past month, and almost 15% received food from community resources three or more times in the past month.

**Table 2:** Description of grocery shopping patterns

	%
Primary grocery store – type of store	
Supermarket (chains, e.g., Food4Less)	80.3
Supermarket (non-chain)	0.0
Ethnic-specialty (e.g., Northgate Gonzalez Market)	14.4
Warehouse clubs/Mass merchandiser-supercenter (e.g., Costco, Walmart Supercenter)	4.5
Other (convenience stores, farmer’s market)	0.8
Distance (in miles) to primary grocery store	
< 1 mile	50.6
1 mile to < 3 miles	29.8
3 + miles	19.6
Number of times visited primary grocery store in past month	
0–1 time	12.0
2–3 times	32.0
4–5 times	30.0
6 + times	26.0
Number of different grocery stores shopped at in past month for major food shopping	
1 store	17.2
2 stores	46.0
3 stores	23.5
4 + stores	13.4
Number of times got food from community resources (e.g., food pantry/ bank, church) in past month	
0 times	64.1
1–2 times	21.3
3 + times	14.6

**Fig. 2:**
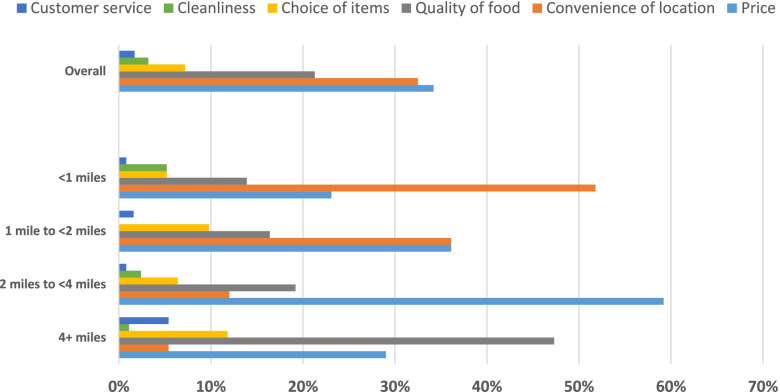
Reason for choosing primary grocery store, overall and by distance travelled

With respect to the main reason for choosing the primary grocery store, the most common reasons were price (34.2%), convenience of location (32.5%), and quality of food (21.3%) (Fig. 2). Others reported that their main reason was choice of items (7.2%), cleanliness (3.2%), or customer service (1.7%). As distance to primary grocery store increased, the percentage of participants who shopped for quality of food increased and the percentage who shopped for convenience of location decreased. Almost half of those who travelled four or more miles cited quality of food as their main reason for choosing a location (47.3%), compared to 13.9% of those who traveled less than one mile. Among those who traveled four or more miles, 5.4% cited convenience of location as the main reason for choosing that location, compared to more than half of those who traveled less than one mile (51.8%). Price was also more commonly cited as the reason among participants who travelled one mile or more than those who traveled less than one mile.

### Regression analyses

Grocery shopping patterns were associated with several participant characteristics (Table 3). Participants who were Hispanic (OR = 0.314, 95% CI 0.181, 0.545) and worked for pay (OR = 0.618, 95% CI 0.383, 0.998) were less likely to shop at a chain Supermarket for their primary grocery shopping. Ordered logit estimates showed participants who were Hispanic (β = 0.555, 95% CI 0.146, 0.965), had a HS/secondary school education or above (β = 0.391, 95% CI 0.013, 0.770), and had access to a car (β = 0.450, 95% CI 0.015, 0.885), were more likely to travel further distances to their primary grocery store. For example, the ordered logit coefficient of close to 0.5 indicates that participants with access to a car and participants who were Hispanic, were approximately 50% more likely than those without access to a car and those who were not Hispanic, respectively, to travel further to their primary grocery store (i.e., traveling 3 + miles compared to traveling 2 miles to < 3 miles or traveling 1 mile to < 2 miles compared to < 1 mile). Similarly, participants with a HS/secondary school education or above were approximately 39% more likely than those with an education below High School/secondary school to travel further to their primary grocery store. Participants at Jordan Downs were less likely to travel further distances (β = -0.740, 95% CI -1.140, -0.340). Participants who were Hispanic (β = 0.443, 95% CI 0.0645, 0.822) were more likely to visit their primary grocery store a greater number of times in the past month. Participants who had access to a car (β = 0.560, 95% CI 0.142, 0.971), or lived at Jordan Downs (β = 0.752, 95% CI 0.374, 1.129) were more likely to visit a greater number of grocery stores in the past month. Participants who had a HS/secondary school education or above (β = -0.522, 95% CI -0.934, -0.111), worked for pay (β = -0.446, 95% CI -0.860, -0.032), obese (β = -0.510, 95% CI -0.887, -0.133), and lived at Jordan Downs (β = -0.476, 95% CI -0.871, -0.082) were less likely to visit a community resource for food. Sensitivity analyses using more detailed categories for independent variables show similar results (Table S1).

**Table 3:** Associations between grocery shopping patterns and participant characteristics

	(1) Primary grocery store was supermarket OR (95% CI)	(2) Distance to primary grocery store Coefficient (95% CI)	(3) # times visited primary grocery store in past month Coefficient (95% CI)	(4) # stores shopped at in past month Coefficient (95% CI)	(5) # times got food from food pantry/bank in past month Coefficient (95% CI)
Age 60 years and older	2.230	-0.205	0.107	-0.384	-0.124
	(0.852, 5.839)	(-0.791, 0.381)	(-0.446, 0.659)	(-0.960, 0.192)	(-0.781, 0.534)
Hispanic	0.314**	0.555**	0.443*	-0.071	-0.229
	(0.181, 0.545)	(0.146, 0.965)	(0.0645, 0.822)	(-0.453, 0.310)	(-0.655, 0.197)
Income $10,000 or higher	1.055	-0.171	0.131	0.307	0.008
	(0.661, 1.685)	(-0.542, 0.200)	(-0.195, 0.456)	(-0.0261, 0.640)	(-0.364, 0.381)
HS/secondary or higher education	0.958	0.391*	0.039	-0.067	-0.522*
	(0.594, 1.544)	(0.0132, 0.770)	(-0.324, 0.401)	(-0.432, 0.299)	(-0.934, -0.111)
Works for pay	0.618*	0.291	-0.0340	0.113	-0.446*
	(0.383, 0.998)	(-0.090, 0.671)	(-0.386, 0.318)	(-0.247, 0.474)	(-0.860, -0.0320)
Has access to car	0.517	0.450*	0.275	0.560**	-0.187
	(0.265, 1.006)	(0.0154, 0.885)	(-0.115, 0.665)	(0.149, 0.971)	(-0.627, 0.252)
Has children under 18 years old living in household	0.773	0.130	0.329	0.173	0.204
	(0.447, 1.336)	(-0.258, 0.519)	(-0.0494, 0.708)	(-0.216, 0.562)	(-0.233, 0.641)
Obese	1.140	-0.0360	0.320	-0.0580	-0.510**
	(0.695, 1.872)	(-0.427, 0.355)	(-0.017, 0.658)	(-0.400, 0.284)	(-0.887, -0.133)
Self-rated diet as very good or excellent	1.217	-0.079	-0.0910	-0.155	-0.092
	(0.757, 1.957)	(-0.462, 0.303)	(-0.436, 0.254)	(-0.504, 0.194)	(-0.482, 0.298)
Lives at Jordan Downs	0.780	-0.740**	0.026	0.752**	-0.476*
	(0.450, 1.352)	(-1.140, -0.340)	(-0.326, 0.377)	(0.374, 1.129)	(-0.871, -0.0820)
N	507	473	507	507	507
AIC BIC Pseudo R^2^	484.5 531.0 0.0815	962.2 1012.1 0.0350	1354.8 1409.7 0.0153	1274.0 1329.0 0.0304	906.9 957.7 0.0275

Logistic regression was estimated for the binary outcome: whether the primary grocery store was a supermarket was the primary grocery store in Column (1). Ordered logit models were estimated in Columns (2) to (5)

^*^p < 0.05, **p < 0.01

The reason for choice of the primary grocery store was associated with several participant characteristics (Table 4). Compared to those who were Non-Hispanic Black, those who were Hispanic were more likely to prioritize price (OR = 2.042, 95% CI 1.292, 3.228), and less likely to prioritize convenience (OR = 0.629, 95% CI 0.408, 0.968). Compared to individuals with a very low household income, those with a yearly household income of $10,000 or more were more likely to prioritize convenience (OR = 1.726, 95% CI 1.151, 2.587), and less likely to prioritize price (OR = 0.666, 95% CI 0.447, 0.992). Individuals with more than a high school education were more likely to prioritize convenience (OR = 1.780, 95% CI 1.154, 2.746), and those with a paying job were more likely to prioritize price (OR = 1.572, 95% CI 1.035, 2.388). Compared to individuals without a car, those with a car were less likely to prioritize convenience (OR = 0.511, 95% CI 0.321, 0.812). Compared to those who were not obese, those with obesity were more likely to prioritize price (OR = 1.528, 95% CI 1.016, 2.298). Those who rated their diet as good, very good or excellent were more likely to prioritize convenience (OR = 1.547, 95% CI 1.005, 2.379). None of the participant characteristics were associated with reporting quality of food or choice of items as the main reason for choosing the primary grocery store. Sensitivity analyses using more detailed categories for independent variables show similar results (Table S2).

**Table 4:** Associations between reasons for primary grocery store choice and participant characteristics

Participant characteristics	Reason for choice of primary grocery store			
	(1) Quality of food OR (95% CI)	(2) Price OR (95% CI)	(3) Choice of items OR (95% CI)	(4) Convenience of Location OR (95% CI)
Age 60 years and older	0.784	0.539*	0.984	1.672
	(0.340, 1.804)	(0.260, 1.116)	(0.241, 4.019)	(0.861, 3.249)
Hispanic	0.769	2.042**	0.539	0.629*
	(0.460, 1.286)	(1.292, 3.228)	(0.212, 1.372)	(0.408, 0.968)
Income $10,000 or higher	0.854	0.666*	1.853	1.726**
	(0.549,1.330)	(0.447, 0.992)	(0.897, 3.831)	(1.151, 2.587)
HS/secondary or higher education	0.903	0.709	0.578	1.780**
	(0.552, 1.475)	(0.463, 1.086)	(0.245, 1.366)	(1.154, 2.746)
Works for pay	0.997	1.572*	0.628	0.769
	(0.617, 1.610)	(1.035, 2.388)	(0.279, 1.415)	(0.497, 1.191)
Has access to car	1.602	1.315	2.421	0.511**
	(0.867, 2.958)	(0.816, 2.121)	(0.786, 7.458)	(0.321, 0.812)
Has children under 18 years old living in household	1.139	0.883	1.622	0.875
	(0.667, 1.944)	(0.568, 1.371)	(0.628, 4.190)	(0.562, 1.363)
Obese	0.919	1.528*	0.933	0.685
	(0.585, 1.443)	(1.016, 2.298)	(0.448, 1.944)	(0.455, 1.033)
Self-rated diet as good, very good or excellent	0.708	0.899	1.031	1.547*
	(0.446, 1.125)	(0.595, 1.357)	(0.491, 2.163)	(1.005, 2.379)
Lives at Jordan Downs	1.096	1.099	0.852	0.898
	(0.673, 1.786)	(0.717, 1.684)	(0.401, 1.809)	(0.586, 1.376)
N	507	507	507	507
AIC BIC Pseudo R^2^	534.7 581.2 0.0139	642.2 688.7 0.0528	270.8 317.4 0.0422	618.1 664.6 0.0682

95% Confidence Interval in parentheses

*p < 0.05, **p < 0.01

## Discussion

Even within low-income, minority communities in Watts, there was considerable variability in households’ grocery shopping patterns, including the types of grocery stores accessed, distance travelled, frequency of shopping, and reasons behind grocery store choice. This study is the first to examine this variability in grocery shopping patterns among women living in public housing and these results are an important addition to the literature for several reasons. First, few studies have examined grocery shopping patterns among low-income groups [46–50], and have offered limited information on the motivations that contribute to these patterns. Public housing communities are an important population to study as they tend to be predominantly low-income, minority women, and have high risk for obesity, inadequate nutrition, and poor food environment in their neighborhood. Second, public housing residents live in clustered housing, resulting in exposure to the same neighborhood food environment. The variability in households’ grocery shopping patterns, despite the similar neighborhood exposure and socioeconomic disadvantage, suggests that grocery shopping patterns are more complex and influenced by factors beyond neighborhood availability. Third, this study provides a benchmark for the grocery shopping patterns of public housing residents in Watts prior to the Jordan Downs redevelopment. As this community undergoes redevelopment, which includes the opening of a new ‘onsite’ supermarket in Jordan Downs, it will be important to examine how residents’ grocery shopping patterns change overtime.

Our findings show that although participants in all three public housing communities had walkable supermarket access, their choices of primary grocery store suggest their needs varied. More than 40 percent of participants shopped beyond the two closest supermarkets, and shopped at more than 50 different stores. Of those shopping beyond the neighborhood, many women shopped at ethnic-specialty stores, suggesting demand for that type of store. Prior qualitative research has noted that Hispanic shoppers often seek out tiendas or ethnic-specialty foods that cater to the population and they are willing to travel for these stores. [25] In addition to seeking ethnic-specialty foods, public housing residents with access to a car may drive out of the neighborhood, rather than walk to the closest supermarket, due to safety concerns in their neighborhood. Although online stores can remove some of the barriers of living in a food desert and lead to healthier dietary choices [51–54], in this study, no participants reported an online store as a primary source for groceries. Use of online grocery shopping is limited amonglow-income groups and individuals utilizing federal assistance due to cost and distrust of the process [51, 52, 55].

In addition to supermarket access, the Watts community had an abundance of small markets and convenience stores. While these are not the stores where participants did their primary grocery shopping, they may be used for quick needs and these types of stores often sell high-caloric foods and little fresh foods [56]. Therefore, the presence of large number of convenience stores may contribute to poor diet, even though the markets are not used for primary grocery shopping.

The findings of this study are in line with qualitative evidence suggesting that low-income families consider many factors, along with price, when making food shopping decisions [26, 30]. Convenience of location was prioritized by an equal number of participants, followed closely by quality of food (prioritized by more than 20 percent of participants). Those that prioritize convenience may not have time or resources to travel further. Price shopping can constrain other resources such as time, which is also commonly a limited commodity among low-income, racial/ethnic minority groups, especially mothers [26, 30]. Interestingly, participants with jobs were more likely to prioritize price than participants without a job (controlling for income). In light of the qualitative evidence from other studies [26, 30], low-income mothers without jobs may not have the resources to travel for cheaper food and may have to shop at whatever store they are closest to. In this sample, women working for pay were more likely to have access to a car and therefore the opportunity (both transportation and child-care) to prioritize price. Participants who traveled the furthest, most often prioritized quality, likely because grocery stores in low-income areas often have more limited options and less fresh foods [12–14], and have to travel further to find better quality and more variety of foods [26].

Another dimension along which there was variability in grocery shopping patterns was race-ethnicity. Differences between Black and Hispanic women explained much of the variability in grocery shopping patterns. Hispanic women were more likely to shop at ethnic-specialty stores (and not chain Supermarkets) and more likely to prioritize their choice of grocery store due to price (100% increase in the odds of shopping for price compared to Black women). Hispanic women were also more likely to travel further distances and shop for groceries more often. These differences in patterns are likely due, in part, to cultural factors including different food preparation and cooking preferences by these groups. Prior qualitative work suggests that Black women are more likely to purchase canned or frozen vegetables because they have preferences for the taste and texture of these products, consider them more convenient, and have limited cooking skills for preparing fresh vegetables, while Hispanic women find fresh fruits and vegetables easier to cook because they are ready to use as is and they learned to cook them from other women in their family [26]. Households that purchase more canned or frozen foods can prioritize convenience over quality and price of fresh foods and do not need to shop for groceries as often. In addition, Hispanic women may prefer to shop at ethnic-specialty stores because although they prefer fresh vegetables, they tend to stick to cooking traditional foods they are familiar with, rather than trying new varieties of vegetables [26].

Finally, our results provide insight into how the introduction of a new ‘onsite’ supermarket might influence public housing residents. On the one hand, an equal number of participants prioritized convenience and price in choosing their primary grocery store and those that prioritize convenience may be more inclined to utilize the new, even more convenient, grocery store. As a result, it is possible that more residents may choose to shop at the new supermarket than in previous studies where residents still shopped at their usual grocery store after new, affordable supermarkets were introduced to their neighborhood [31–33]. On the other hand, other residents may not shop at the new neighborhood supermarket unless the store fulfils residents’ needs, such as offering food that is as affordable and high quality, at least as well as their previous primary grocery shopping location. Participants that shop at ethnic-specialty stores, which are not already available in Watts, will likely continue to travel outside of Watts to those stores if the new supermarket does not stock the desired foods. Future waves of data from the Watts Neighborhood Health Study will allow us to examine changes in grocery shopping patterns after the supermarket opening.

Our findings should be interpreted in light of its limitations. First, the cross-sectional nature of the analysis limits the ability to determine causality. Second, this study focused on low-income, racial and ethnic minority women living in public housing communities in South Los Angeles. As such, the results of this study may not be generalizable to other contexts outside urban public housing communities in the U.S.. For example, in communities not dominated by public housing, residents are not as clustered so there may be more variability in food environments. Additionally, women who have higher income may have different priorities for grocery shopping, as they are less likely to be hindered by restricted food access; therefore, grocery shopping patterns may influence their dietary patterns less than other factors such as palatability and preferences for food. Third, participants were recruited as the head of the household and/or main caregiver, but were not recruited specifically as the primary grocery shopper. Finally, we only examined the households’ primary grocery shopping location, which does not capture all of their grocery shopping behaviors or priorities.

## Conclusions

This study contributes to the growing body of evidence on the complex and interactional nature of individuals’ behaviors with social-ecological influences within their broader environment [9]. The results indicate that, even *within* low-income, minority communities, there is considerable variation in households’ grocery shopping patterns, including the types of grocery stores accessed, distance travelled, frequency of shopping, and reasons behind grocery store choice, and that these patterns were associated with individual and household characteristics. The results of this study and others [21, 57] suggest that initiatives to improve the neighborhood food environment should consider quality of food, cultural factors, and availability of foods desired by the surrounding community, in addition to price and proximity of grocery stores. These findings can inform policies that seek to address the diverse food shopping needs and preferences of low-income families, instead of one-size-fits all approaches.

Future research on the link between the neighborhood food environment and diet should consider the complexity of how low-income households navigate and interact with their food environment. Additionally, future research with this study population will consider what types of foods are being purchased at one’s primary grocery location, as well as other food outlets, to determine how the grocery shopping patterns identified in this study contribute to dietary quality.

## Supplementary Information

**Additional file 1: Table S1.** Associations between grocery shopping patterns and participant characteristics. **Table S2.** Associations between reasons for primary grocery store choice and participant characteristics.

## Data Availability

The datasets generated and/or analysed during the current study are not publicly available due to ongoing data analysis and identifiable data but are available from the corresponding author on reasonable request.
