# Supplementary material for: Heterogeneity in grocery shopping patterns among low-income minority women in public housing
Source: BMC Public Health. 2022 Aug 24;22:1612. doi: 10.1186/s12889-022-14003-0 (PMC9404610; doi:10.1186/s12889-022-14003-0)
Supplement: Supplementary file 1 — Additional file 1: Table S1. Associations between grocery shopping patterns and participant characteristics. Table S2. Associations between reasons for primary grocery store choice and participant characteristics. [file 12889_2022_14003_MOESM1_ESM.docx]

Table S1: Associations between grocery shopping patterns and participant characteristics

|  | (1)  Primary grocery store was supermarket  OR (95% CI) | (2)  Distance to primary grocery store  Coefficient  (95% CI) | (3)  # times visited primary grocery store in past month  Coefficient  (95% CI) | (4)  # stores shopped at in past month  Coefficient  (95% CI) | (5)  # times got food from food pantry/bank in past month  Coefficient  (95% CI) |
| --- | --- | --- | --- | --- | --- |
| Age | 1.644 | 0.076 | -0.031 | 0.420** | 0.142 |
| 35-59 | (0.931, 2.901) | (-0.369, 0.521) | (-0.442, 0.381) | (0.0218, 0.818) | (-0.302, 0.585) |
| 60+ | 3.616* | -0.148 | 0.090 | 0.081 | -0.103 |
|  | (1.093, 11.97) | (-0.857, 0.562) | (-0.573, 0.753) | (-0.576, 0.738) | (-0.918, 0.712) |
| Hispanic | 0.316** | 0.484* | 0.448* | -0.001 | -0.263 |
|  | (0.171, 0.584) | (0.0384, 0.930) | (0.0421, 0.854) | (-0.400, 0.399) | (-0.704, 0.179) |
| Household Income  $5,000 - $9,999 | 0.595 | -0.164 | 0.0922 | 0.116 | 0.440 |
|  | (0.301, 1.174) | (-0.689, 0.361) | (-0.351, 0.535) | (-0.378, 0.610) | (-0.0985, 0.979) |
| $10,000– $14,999 | 1.201 | -0.317 | 0.024 | 0.255 | 0.390 |
|  | (0.548, 2.631) | (-0.860, 0.227) | (-0.506, 0.555) | (-0.238, 0.748) | (-0.144, 0.924) |
| $15,000 or more | 0.711 | -0.242 | 0.297 | 0.348 | -0.0134 |
|  | (0.388, 1.306) | (-0.697, 0.214) | (-0.0990, 0.693) | (-0.0604, 0.756) | (-0.504, 0.477) |
| Education | 1.066 | -0.029 | 0.100 | 0.499 | -0.214 |
| Some high school/ secondary | (0.543, 2.094) | (-0.605, 0.547) | (-0.401, 0.600) | (-0.0298, 1.028) | (-0.754, 0.327) |
| High school/secondary/GED | 1.257 | 0.195 | 0.261 | 0.265 | -0.734* |
|  | (0.630, 2.510) | (-0.333, 0.722) | (-0.220, 0.742) | (-0.271, 0.802) | (-1.320, -0.147) |
| More than high school/secondary | 1.010 | 0.661* | -0.196 | 0.392 | -0.441 |
|  | (0.463, 2.205) | (0.108, 1.214) | (-0.779, 0.386) | (-0.178, 0.962) | (-1.075, 0.192) |
| Works for pay | 0.624 | 0.233 | -0.020 | 0.146 | -0.424* |
|  | (0.380, 1.025) | (-0.153, 0.619) | (-0.383, 0.343) | (-0.233, 0.524) | (-0.846, -0.00236) |
| Has access to car | 0.545 | 0.545* | 0.265 | 0.552** | -0.255 |
|  | (0.274, 1.087) | (0.106, 0.983) | (-0.114, 0.644) | (0.134, 0.971) | (-0.716, 0.206) |
| Has children under 18 years old living in household | 0.847 | 0.115 | 0.309 | 0.208 | 0.236 |
|  | (0.474, 1.516) | (-0.299, 0.530) | (-0.091, 0.709) | (-0.205, 0.621) | (-0.230, 0.701) |
| Obese | 1.168 | -0.051 | 0.314 | -0.063 | -0.502** |
|  | (0.714, 1.911) | (-0.440, 0.339) | (-0.0363, 0.664) | (-0.408, 0.282) | (-0.881, -0.123) |
| Self-rated diet  Good | 0.714 | 0.137 | 0.054 | 0.224 | 0.107 |
|  | (0.407, 1.252) | (-0.300, 0.574) | (-0.388, 0.496) | (-0.218, 0.666) | (-0.347, 0.561) |
| Very good or excellent | 0.907 | 0.074 | 0.171 | 0.128 | -0.0003 |
|  | (0.426, 1.932) | (-0.523, 0.670) | (-0.331, 0.673) | (-0.340, 0.596) | (-0.570, 0.569) |
| Lives at Jordan Downs | 0.722 | -0.451* | 0.0150 | 0.720** | -0.457* |
|  | (0.410, 1.271) | (-0.849, -0.053) | (-0.338, 0.368) | (0.309, 1.132) | (-0.847, -0.067) |
| N | 507 | 473 | 507 | 507 | 507 |
| AIC  BIC  Pseudo R^2^ | 487.8  559.7  0.0987 | 1150.2  1229.2  0.0262 | 1361.6  1441.9  0.0192 | 1278.2  1358.5  0.0365 | 912.4  988.5  0.0347 |
| Note: Logistic regression was estimated for the binary outcome: whether the primary grocery store was a supermarket was the primary grocery store in Column (1). Ordered logit models were estimated in Columns (2) to (5)  *p<0.05, **p<0.01 | | | | | |

Table S2: Associations between reasons for primary grocery store choice and participant characteristics

|  | Reason for choice of primary grocery store | | | |
| --- | --- | --- | --- | --- |
|  | (1)  Quality of food  OR (95% CI) | (2)  Price  OR (95% CI) | (3)  Choice of items  OR (95% CI) | (4)  Convenience of Location  OR (95% CI) |
| Age | 1.326 | 0.541* | 2.659 | 0.945 |
| 35-59 | (0.737, 2.386) | (0.333, 0.880) | (0.961, 7.356) | (0.572, 1.561) |
| 60+ | 0.939 | 0.299** | 2.357 | 1.763 |
|  | (0.341, 2.586) | (0.122, 0.733) | (0.430 ,12.90) | (0.770, 4.037) |
| Hispanic | 0.776 | 1.960** | 0.583 | 0.611* |
|  | (0.451, 1.335) | (1.225, 3.134) | (0.208, 1.631) | (0.388, 0.962) |
| Household Income  $5,000 - $9,999 | 1.645 | 0.805 | 1.010 | 1.077 |
|  | (0.878, 3.083) | (0.469, 1.382) | (0.309, 3.301) | (0.595, 1.949) |
| $10,000– $14,999 | 1.129 | 0.559 | 2.607 | 1.360 |
|  | (0.566, 2.249) | (0.301, 1.039) | (0.997, 6.821) | (0.732, 2.525) |
| $15,000 or more | 1.014 | 0.687 | 1.266 | 2.008** |
|  | (0.565, 1.819) | (0.417, 1.131) | (0.485, 3.307) | (1.199, 3.362) |
| Education | 1.023 | 0.778 | 1.088 | 1.288 |
| Some high school/ secondary | (0.523, 2.000) | (0.439, 1.379) | (0.398, 2.977) | (0.678, 2.448) |
| High school/secondary/GED | 1.093 | 0.547* | 0.798 | 1.751 |
|  | (0.561, 2.131) | (0.305, 0.980) | (0.260, 2.450) | (0.946, 3.241) |
| More than high school/secondary | 0.806 | 0.471* | 0.673 | 2.511** |
|  | (0.356, 1.822) | (0.235, 0.943) | (0.170, 2.668) | (1.254, 5.030) |
| Works for pay | 1.066 | 1.484 | 0.762 | 0.714 |
|  | (0.652, 1.745) | (0.977, 2.253) | (0.332, 1.750) | (0.452, 1.128) |
| Has access to car | 1.476 | 1.359 | 2.336 | 0.517** |
|  | (0.789, 2.762) | (0.835, 2.211) | (0.761, 7.176) | (0.322, 0.832) |
| Has children under 18 years old living in household | 1.189 | 0.789 | 1.976 | 0.853 |
|  | (0.677, 2.087) | (0.502, 1.239) | (0.738, 5.293) | (0.537, 1.354) |
| Obese | 0.937 | 1.515* | 1.023 | 0.657* |
|  | (0.592, 1.483) | (1.002, 2.290) | (0.492, 2.126) | (0.434, 0.996) |
| Self-rated diet  Good | 1.167 | 1.288 | 0.521 | 0.842 |
|  | (0.653, 2.084) | (0.794, 2.090) | (0.162, 1.679) | (0.501, 1.412) |
| Very good or excellent | 1.748 | 0.922 | 1.390 | 0.463* |
|  | (0.931, 3.281) | (0.491, 1.733) | (0.579, 3.340) | (0.249, 0.861) |
| Lives at Jordan Downs | 1.083 | 1.144 | 0.804 | 0.899 |
|  | (0.654, 1.796) | (0.740, 1.769) | (0.394, 1.640) | (0.580, 1.395) |
| N | 507 | 507 | 507 | 507 |
| AIC  BIC  Pseudo R^2^ | 540.8  612.7  0.0251 | 645.4  717.2  0.0662 | 273.2  345.1  0.0792 | 623.1  695.0  0.0792 |
| Note: Logistic regression was estimated for the binary outcome: whether the primary grocery store was a supermarket was the primary grocery store in Column (1). Ordered logit models were estimated in Columns (2) to (5)  *p<0.05, **p<0.01 | | | | |
